# Utilization patterns of Chinese medicine and Western medicine under the National Health Insurance Program in Taiwan, a population-based study from 1997 to 2003

**DOI:** 10.1186/1472-6963-8-170

**Published:** 2008-08-09

**Authors:** Lee-Chin Chang, Nicole Huang, Yiing-Jenq Chou, Chen-Hua Lee, Feng-Yu Kao, Yi-Tsau Huang

**Affiliations:** 1 Institute of Traditional Medicine & Institute of Public Health, School of Medicine, National Yang Ming University, Taipei, Taiwan; 2 Department of Social Medicine & Institute of Public Health, School of Medicine, National Yang Ming University, Taipei, Taiwan; 3 Bureau of National Health Insurance, Taipei, Taiwan; 4 Committee on Chinese Medicine and Pharmacy, Department of Health, Taipei, Taiwan

## Abstract

**Background:**

In 1995, Taiwan has launched a national health-care system (the National Health Insurance Program, NHI) covering the use of both Western medicine (WM) and Chinese medicine (CM). This population-based study was conducted to understand the role of CM in this dual medical system by determining the utilization patterns of CM and WM and to analyze the demographic characteristics and primary indications influencing the choice of the medical services for the development of strategies to enhance the appropriate use and reduce unnecessary use of CM.

**Methods:**

This study used the NHI sample files from 1997 to 2003 consisting of comprehensive utilization and enrolment information for a random sample of 200,432 NHI beneficiaries of the total enrolees from 1995 to 2000. A total of 136,720 subjects with valid and complete enrolment and utilization data were included in this study. The logistic regression method was employed to estimate the odds ratios (ORs) for utilization of CM and WM. The usage, frequency of services, and primary indications for CM and WM were evaluated. A significance level of α = 0.05 was selected.

**Results:**

Compared with WM, the odds of CM increased from 1997 to 2003. The odds of using CM (OR = 1.48; 95% CI: 1.45–1.50; p < 0.001) and WM (OR = 1.74; 95% CI: 1.72–1.77; p < 0.001) were higher in females and that of CM increased with age to a peak in the 45–54-year-group (OR = 1.75; 95% CI: 1.68–1.82; p < 0.001) and WM (OR = 1.09; 95% CI: 1.05–1.13; p < 0.001) in the elderly subjects (≥ 65 years). The odds of CM and WM were similar in all income groups. However, those of CM were higher in Central (OR = 1.65; 95% CI: 1.56–1.74; p < 0.001) and Southern Taiwan (OR = 1.18; 95% CI: 1.12–1.25; p < 0.001) and lower in the remote areas (OR = 0.57; 95% CI: 0.52–0.63; p < 0.001). Most of the patients had one ambulatory visit of both medical services annually. However, the utilization of WM predominated over CM. Over 90% of CM service was provided by clinics, whereas over 60% of WM service by hospitals. Diseases of the respiratory system was the most frequent primary indication in CM and WM. Herbal medication was the most commonly used form of CM (68.4–72.7%).

**Conclusion:**

In recent years, there is an increasing trend in the utilization of CM in Taiwan. This increasing trend may be due to the covering of CM in the national health insurance system.

## Background

There is an increasing trend in the utilization of complementary or alternative medicine (CAM) worldwide [1]. According to the estimation of the World Health Organization, the usage of CAM ranges from 9 to 65% in different countries [2]. In the United States, CAM utilization increased from 34% in 1990 to 42% in 1997 [3,4]. CAM is also very popular in Europe [5], Canada [6], and Australia [7]. Patients choosing CAM are not necessarily dissatisfied with conventional medical care. They are seeking more effective ways to improve their health and well-being and to relieve symptoms associated with chronic, even terminal, illnesses or the side effects of conventional treatments for them [8].

Among different forms of CAM, Chinese medicine (CM) is well known for the medicinal formulas and acupuncture, and is one of the most popular alternative medicines in many countries. In Singapore, CM is the most popular form of CAM and has been employed by 88% of the CAM users [9]. Moreover, CM accounts for 40% of the health care in China [10]. Although CM is commonly used in the countries of East Asia, this system of medical service is also growing in popularity and offers an important alternative or complement to biomedical care in the Western countries [11].

In Taiwan, one distinguishing feature of the national health-care insurance system is the co-existence of the modern Western medicine (WM) and CM. In addition, the social health-care insurance system covers CM since 1975. Although only bone fractures and dislocations were included in the initial coverage of the Labor Medical Insurance, the therapeutic items were then expanded to internal medicine, gynecology, and acupuncture in 1983. In 1988, the Public Employee Medical Insurance Program started to reimburse CM [12]. After the implementation of the National Health Insurance (NHI) Program in March 1995, all citizens who have established a registered domicile in Taiwan are mandated to join the program. In June 2003, more than 99% of the 23-million population has benefited from this universal health insurance program. However, CM has only 5% of the total expenditure in the NHI coverage. The health care items covered physician consultation for diagnosis, drug prescription, acupuncture, and muscle strain therapy [13].

Although information on different aspects of CM in Taiwan is available [12,14-18], these reports were based on small samples [12,14-16] or limited to one component of CM [17,18]. Moreover, some of these studies were conducted before the establishment of the NHI program [12,14,15]. Recently, a study on the use frequencies, characteristics of users, and the disease categories treated by CM in Taiwan was conducted based on the complete datasets of CM outpatient reimbursement claims from 1996 to 2001 [19]. However, except sex and age, other user characteristics were not analyzed and the utilization pattern of WM was only slightly explored. The purpose of this study is to gain a more complete picture of utilization of CM in the national health insurance system with dual medical systems (Western and Chinese medical services) by determining the extent of CM utilization from 1997 to 2003 using WM as a reference and the demographic factors and primary indications that predict the choice of the type of medical service.

## Methods

### Data Resource

Since all citizens in Taiwan who have established a registered domicile should be enrolled in the NHI Program, the NHI Bureau has accumulated 23.75 million administrative and claims records, forming the largest such collection in the world. The NHI research database was established under the cooperation of National Health Research Institutes (NHRI) and the NHI Bureau [13]. The NHRI safeguards the privacy and confidentiality of the subjects [20]. The database includes the NHI claims data and data from the enrolment and provider files. In addition to birth date and sex of each patient, the NHI claims data also record diagnosis, date of service, drugs prescribed and filed, dispensing method and anonymous identifiers for the patient, the hospital/clinic and the physician providing the service. All the individuals included in the entire claims database (the general population) were given different random numbers by using a random number function. Simple random sampling of about 50,000 people at a time was performed in 2000. This sampling database consisted of four simple randomly sampled subsets and finally included 200,432 enrolees and represents 1% of the total NHI beneficiaries. [20,21].

### Study Sample

In this study, a nationally representative sample of 200,432 NHI enrollees was used. To construct a fixed cohort, we excluded those who were newborns aged below 2 years (5.0%) in 1997, died during the study period (3.4%), were foreigners (4.1%), had incomplete data (1.0%), and had no continuous enrollment information from 1997 to 2003 (18.2%) sequentially. The final fixed cohort consisted of 136,720 individuals. These individuals were observed to investigate the longitudinal utilization patterns of CM and WM from 1997 to 2003.

### Study variables

In order to understand the common factors affecting the utilization of CM and WM, we selected the demographic factors according to previous studies [12,22,23]. In addition to gender, ages were categorized into eight groups: 2–7, 8–14, 15–24, 25–34, 35–44, 45–54, 55–64 and 65 years or older. For the socio-economic status (SES), we classified those with a well-defined monthly wage into three categories: ≥ US$1,280, US$640–1,279 and < US$640. Those people without a well-defined monthly wage were categorized into two groups: farmers and fishermen, and others, which include veterans, low-income people and individuals enrolled in the NHI through local government offices. Severe diseases were defined according to the Illness and Injury Severity Score and the NHI has an official list of major diseases including cancer, acquired immune deficiency syndrome, major psychiatric disorder, and so on [13]. Remote areas were defined officially according to mountainous geographic environment and traffic conditions. There are 30 mountainous regions proclaimed remote areas [21]. In addition, offshore islands include Lanyu Isles, Green Island, and Penghu Islands. These remote areas have been under-served with insufficient medical services.

### Definition of CM

In this study, CM is used pragmatically to refer to diagnostic and therapeutic practices, including primarily herbal medication, acupuncture, and muscle strain therapy. Muscle strain therapy includes professional massage and osteopathy manual therapy. In this study, we also considered dislocation treatment as one component of the muscle strain therapy. These three components of CM are also modalities of CAM defined in the Western countries [3]. In addition to these three main components, CM users may also consult the physician only for physical examination or suggestion of management of the health care problems but without treatment. Consultation services were also covered by the NHI Program.

### Statistical analysis

The unit of observation was each individual in the study sample. The usage, frequency of services, and primary indications for CM and WM were evaluated. The classification of primary indications was according to the International Classification of Diseases Ninth Clinical Modification (ICD-9-CM) [25]. The statistical software SAS 9.13 [26] was used for data management and analyses. A logistic regression model was used to analyze the data, and generalized estimating equations were used to account for correlation among the repeated measurements [27,28]. Based on the correlation matrices of CM and WM utilizations over time, an unstructured working correlation was selected. In addition, sensitivity analyses were conducted for autoregressive, independent and compound symmetry, and the results remained robust. A significance level of α = 0.05 was selected.

## Results

Table 1 shows the demographic characteristics of the studied cohort. Adjusted ORs and 95% confidence intervals (95% CIs) resulting from the logistic regression model are displayed in Table 2. The odds of using CM (OR 1.48) and WM (OR 1.74) were higher for females than for males (OR 1.00). Compared with the youngest age group (OR 1.00), the odds of CM increased with age to a peak in the 45–54-year-group (OR 1.75) whereas those of WM became relatively high only in the elderly subjects (≥ 65 years) (OR 1.09).

**Table 1:** Demographic characteristics of the studied cohort (*n *= 136,720)

Characteristic*	No. of subjects	%
Gender		
Male	68,135	49.8
Female	68,585	50.2
Age (years)		
2–7	9,648	7.1
8–14	11,933	8.7
15–24	23,244	17.0
25–34	26,903	19.7
35–44	27,307	20.0
45–54	17,273	12.6
55–64	9,986	7.3
≥ 65	10,426	7.6
Socio-economic status		
< US$640	54,729	40.0
US$640-1279	29,788	21.8
≥ US$1280	11,137	8.1
Farmers and fishermen	21,568	15.8
Others	19,498	14.3
Severe disease		
Without	132,835	97.2
With	3,885	2.8
Region		
Taipei	43,392	31.7
Northern Taiwan	18,830	13.8
Central Taiwan	26,732	19.6
Southern Taiwan	21,241	15.5
Kaohsiung and Pingtung	22,848	16.7
East Taiwan	3,677	2.7
Remote Area		
Mountainous regions	1,159	0.9
Offshore islands	1,156	0.9
General Population	134,405	98.2

*Determined from 1997 prospectively.

**Table 2:** Adjusted odds ratios and 95% confidence intervals for characteristics associated with the utilization of Chinese medicine and Western medicine (*n *= 136,720)

Characteristic	Chinese medicine	*p *value	Western medicine	*p *value
Gender				
Male	1.00		1.00	
Female	1.48 (1.45–1.50)*	< 0.001	1.74 (1.72–1.77)	< 0.001
Age (years)				
2–7	1.00		1.00	
8–14	1.14 (1.10–1.18)	< 0.001	0.50 (0.49–0.51)	< 0.001
15–24	1.38 (1.33–1.44)	< 0.001	0.36 (0.36–0.37)	< 0.001
25–34	1.58 (1.52–1.64)	< 0.001	0.38 (0.37–0.38)	< 0.001
35–44	1.74 (1.67–1.81)	< 0.001	0.39 (0.38–0.40)	< 0.001
45–54	1.75 (1.68–1.82)	< 0.001	0.45 (0.44–0.46)	< 0.001
55–64	1.63 (1.56–1.71)	< 0.001	0.64 (0.63–0.66)	< 0.001
≥ 65	1.51 (1.44–1.58)	< 0.001	1.09 (1.05–1.13)	< 0.001
Socioeconomic status				
≥ US$1,280	1.00		1.00	
US$640-1,279	1.05 (1.03–1.07)	< 0.001	1.00 (0.98–1.01)	0.473
< US$640	1.04 (1.02–1.06)	< 0.001	0.98 (0.96–0.99)	< 0.001
Farmers and fishermen	0.90 (0.87–0.92)	< 0.001	0.97 (0.95–0.99)	< 0.001
Others	0.86 (0.84–0.88)	< 0.001	0.77 (0.76–0.78)	< 0.001
Severe disease				
Without	1.00		1.00	
With	1.00 (0.95–1.05)	0.900	2.40 (2.29–2.52)	< 0.001
Region				
Eastern Taiwan	1.00		1.00	
Taipei	0.91 (0.86–0.96)	0.001	1.03 (0.99–1.07)	0.117
Northern Taiwan	0.96 (0.90–1.01)	0.123	1.08 (1.04–1.13)	< 0.001
Central Taiwan	1.65 (1.56–1.74)	< 0.001	1.15 (1.11–1.20)	< 0.001
Southern Taiwan	1.18 (1.12–1.25)	< 0.001	1.20 (1.15–1.25)	< 0.001
Kaohsiung and Pingtung	1.09 (1.03–1.16)	0.002	1.21 (1.16–1.26)	< 0.001
Remote Area				
General Population	1.00		1.00	
Mountainous regions	0.57 (0.52–0.63)	< 0.001	1.03 (0.96–1.11)	0.356
Offshore islands	0.78 (0.70–0.86)	< 0.001	1.21 (1.13–1.30)	< 0.001
Season				
Winter (December – February)	1.00		1.00	
Spring (March – May)	0.95 (0.95–0.96)	< 0.001	0.88 (0.88–0.89)	< 0.001
Summer (June – August)	0.90 (0.89–0.90)	< 0.001	0.79 (0.79–0.80)	< 0.001
Autumn (September – November)	0.93 (0.93–0.94)	< 0.001	0.86 (0.85–0.86)	< 0.001
Year				
1997	1.00		1.00	
1998	1.05 (1.03–1.06)	< 0.001	1.10 (1.10–1.11)	< 0.001
1999	1.10 (1.09–1.12)	< 0.001	1.21 (1.20–1.22)	< 0.001
2000	1.10 (1.09–1.11)	< 0.001	1.26 (1.25–1.27)	< 0.001
2001	1.11 (1.09–1.12)	< 0.001	1.27 (1.26–1.28)	< 0.001
2002	1.12 (1.10–1.13)	< 0.001	1.28 (1.26–1.29)	< 0.001
2003	1.15 (1.14–1.17)	< 0.001	1.17 (1.16–1.19)	< 0.001

*Odds ratio (95% confidence interval).

The odds of CM (OR 1.05) and WM (OR 1.00) in the group with a monthly wage US$640-1,279 were higher than those in the low-income group (< US$640) (CM: OR 1.04; WM: OR 0.98), farmers and fishermen (CM: OR: 0.90; WM: OR 0.97) and the other group (CM: OR 0.86; WM: OR: 0.77). Although the odds of CM were similar in the groups with (OR 1.00) and without (OR 1.00) severe diseases, the odds of WM in the people with severe diseases (OR 2.40) was higher than those without these diseases (OR 1.00) (Table 2).

The odds of CM were higher among people in Central (OR 1.65), Southern (OR 1.18), and Kaohsiung and Pingtung (OR 1.09) than those in Eastern Taiwan (OR 1.00), Northern Taiwan (OR 0.96), and Taipei (OR 0.91). However, those of WM were higher among people in Taipei (OR 1.03), Northern Taiwan (OR 1.08), Central Taiwan (OR 1.15), Southern Taiwan (OR 1.20), and Kaohsiung and Pingtung (OR 1.21) than Eastern Taiwan (OR 1.00). The odds of CM in the general population (OR 1.00) was higher than those in the mountainous regions (OR 0.57) and offshore islands (OR 0.78) whereas those of WM were higher in mountainous regions (OR 1.03) and offshore islands (OR 1.21) than the general population (OR 1.00) (Table 2).

Table 2 also shows a steady increasing trend in the odds of CM from 1997 (OR 1.00) to 2003 (OR 1.15). Moreover, the odds WM utilization also increased from 1997 (OR 1.00) to 2002 (OR 1.28) but decreased in 2003 (OR 1.17). Of all seasons, the odds of either CM or WM were the highest in winter.

The crude utilizations of CM and WM are shown in Table 3. The number of CM users increased from 36,372 in 1997 to 41,823 in 2003. However, the number of WM users from 115,833 in 1997 to 121,605 in 2002 and decreased to 120,926 in 2003. The average number of patients who had used any CM service in one year and the number of visits to CM providers were 39,562 and 191,612, respectively. The corresponding figures for WM were 119,915 and 1,542,342. Most of the patients had one ambulatory visit a year. However, the utilization frequency of WM (mean = 12.55–13.34, median = 8–9) was higher than that of CM (mean = 4.63–5.03, median = 2–3). Most of the ambulatory CM service was provided by clinics (93.4–96.1%) and only 3.9–6.6% was provided by hospitals. Moreover, the frequency of ambulatory visits in hospitals steadily increased from 3.9% in 1997 to 6.6% in 2002. On the other hand, clinics provided about two-thirds (60.5–67.8%) of the ambulatory WM service, outpatient department of the hospitals took the remaining one-third (32.2–39.5%) of ambulatory service.

**Table 3:** Crude utilizations of Chinese and Western medicine under the NHI program, 1997–2003 (total population = 136,720)

			Frequency of utilization			Provider	
				
Year	No. of users	No. of visits	Mean ± SD	Median	Mode	Hospital (%)	Clinic (%)
Chinese medicine							
1997	36,372	181,109	4.98 ± 6.59	3	1	7,060 (3.9)	174,049 (96.1)
1998	37,622	189,330	5.03 ± 6.60	3	1	7,709 (4.1)	181,621 (95.9)
1999	39,635	196,908	4.97 ± 6.36	3	1	9,461 (4.8)	187,447 (95.2)
2000	40,227	188,713	4.69 ± 5.81	2	1	10,330 (5.5)	178,383 (94.5)
2001	40,425	187,198	4.63 ± 5.73	3	1	10,865 (5.8)	176,333 (94.2)
2002	40,833	191,251	4.68 ± 5.82	3	1	12,617 (6.6)	178,634 (93.4)
2003	41,823	206,777	4.94 ± 6.45	3	1	11,497 (5.6)	195,280 (94.4)
Western medicine							
1997	115,833	1,486,570	12.83 ± 13.82	8	1	478,493 (32.2)	1,008,077 (67.8)
1998	118,504	1,553,383	13.11 ± 14.07	9	1	509,743 (32.8)	1,043,640 (67.2)
1999	120,362	1,606,111	13.34 ± 13.85	9	1	549,420 (34.2)	1,056,691 (65.8)
2000	120,982	1,552,771	12.83 ± 12.92	9	1	554,656 (35.7)	998,115 (64.3)
2001	121,190	1,533,878	12.66 ± 12.97	9	1	583,055 (38.0)	950,823 (62.0)
2002	121,605	1,545,956	12.71 ± 13.23	9	1	610,399 (39.5)	935,557 (60.5)
2003	120,926	1,517,722	12.55 ± 13.69	8	1	568,128 (37.4)	949,594 (62.6)

Diseases of the respiratory system (CM 22.1%, WM 35.6%) and the musculoskeletal system and connective tissue (CM 18.1%, WM 7.5%) were the top two primary indications in the ambulatory health care of both CM and WM. In CM, the remaining common primary indications were injury and poisoning (16.2%), diseases of signs, symptoms and ill-defined conditions (14.2%), and diseases of the digestive system (11.4%). In WM, diseases of the genitourinary system (7.2%), diseases of the digestive system (7.2%), and diseases of sense organs (7.1%) were also common primary indications (Table 4).

**Table 4:** Primary indications in ambulatory visits of Chinese and Western medicine under the National Health Insurance Program in Taiwan from 1997 to 2003

Primary indication (ICD-9-CM code)	Chinese medicine (*n*=1,341,286)	Western medicine (*n*=10,796,391)
1 Infectious and parasitic diseases	0.5*	2.3
2 Malignant neoplasms	0.2	0.7
3 Other neoplasms	0.1	0.7
4 Endocrine, nutritional and metabolic diseases and immunity disorders	1.3	3.8
5 Mental disorders	0.6	2.0
6 Diseases of the nervous system	1.6	1.3
7 Diseases of the sense organs	1.2	7.1
8 Diseases of the circulatory system	1.8	6.3
9 Diseases of the respiratory system	22.1	35.6
10 Diseases of the digestive system	11.4	7.2
11 Diseases of the genitourinary system	7.2	7.2
12 Complications of pregnancy, childbirth and the puerperium	0.1	0.4
13 Diseases of skin and subcutaneous tissue	3.1	6.2
14 Diseases of the musculoskeletal system and connective tissue	18.1	7.5
15 Congenital anomalies	0.1	0.1
16 Certain conditions originating in the perinatal period	0.0	0.0
17 Signs, symptoms and ill-defined conditions	14.2	3.7
18 Injury and poisoning	16.2	4.5
19 Accidents and self inflicted injury	0.0	0.1
20 Other reasons for contact with health services	0.2	3.3

*%.

Herbal medication was the most important component of CM. This component accounted for more than two-thirds of the ambulatory visits (68.4–72.7%). The utilization rate decreased from 70.8% in 1997 to 68.7% in 2003. The other two major components were muscle strain therapy (including dislocation therapy) and acupuncture. Muscle strain therapy accounted for 15.6–17.5% and acupuncture for 9.2–13.0% of the ambulatory visits. These two components showed increasing utilization trends: muscle strain therapy increased from 16.4% in 1997 to 17.2% in 2003 and acupuncture from 9.4% in 1997 to 13.0% in 2003. In addition to these main components of CM, general consultation services accounted for 3.4% in 1997. However, the utilization of these consultation only services decreased to 1.1% in 2003 (Table 5).

**Table 5:** Percentage distribution of ambulatory visits for different components of Chinese medicine under the National Health Insurance Program in Taiwan from 1997 to 2003

	Year						
	
Component*	1997	1998	1999	2000	2001	2002	2003
Herbal medication	70.8	72.7	71.8	70.5	69.3	68.4	68.7
Muscle strain therapy (Including dislocation therapy)	16.4	15.6	15.6	16.0	16.6	17.5	17.2
Acupuncture	9.4	9.2	10.0	11.5	12.1	12.9	13.0
Consultation only and others	3.4	2.5	2.6	2.0	2.0	1.2	1.1

Total	179,464	205,637	221,440	206,108	208,487	210,720	225,705

*More than one component may be provided in each visit.

## Discussion

In this study, the logistic regression method was used to identify patient characteristics associated with the utilization patterns of CM and WM over time. Females used both health care services more than males. The higher utilization of CM in females has also been reported in Singapore [9] and the Western countries [11,29-32] as well as in Taiwan [12,14,16,19]. In practice, CAM has been used to treat postpartum conditions, menopause, and chronic diseases among women [16,19,29,32-35]. Moreover, the age distribution of CM utilization peaked at 45–54 years. This finding is similar to those reported previously [11,12,16,19,29-32]. Since WM provides health-care services such as vaccinations for the children [36], the utilization was revealed to increase with age and peak in youngest group (2–7 years). Moreover, WM utilization was highest in the elderly people (≥ 65 years).

The utilization of CM was higher in the regular salary income group than the low-income group, farmers and fishermen and the other group. These results are similar to the previous findings that CAM users are those with higher education and in the middle to upper socioeconomic status [3,4,37,38]. WM was more frequently used by patients with severe diseases. These diseases require long-term supportive treatment and may not be suitable for CM. Although CM usage was more prevalent in the central and southern parts of Taiwan, CM was not frequently used in mountainous areas and offshore islands. One possible explanation for this phenomenon is the uneven distribution of CM providers, since there are only 6 CM hospitals and 24 CM clinics in eastern Taiwan [13].

In a previous study, 62.5% of the valid beneficiaries in the NHI Program have been reported to use CM at least once from 1996 to 2001 [19]. By controlling the co-variant factors, we found an increasing trend in the utilization of CM and WM. Moreover, 39,562 patients used CM services in each year whereas the corresponding figures for WM were 119,915. Most of patients had a single ambulatory visit annually. However, the mean utilization frequency of WM (12.55–13.34) was higher than that of CM (4.63–5.03). The numbers reported in this study may seem higher than those reported previously [19] because these values were based on only users, not total population. In addition, we revealed that over 90% of the CM services were provided by clinics whereas clinics only provided about two-thirds (60.5–67.8%) of the ambulatory WM services. These findings were consistent with those reported previously [15,19,39]. Under the NHI Program, copayments vary with the provider type. They are highest for ambulatory health care at medical centres and lowest for clinics [40]. Moreover, the copayments in CM are lower than WM. Therefore, more than 90% of the CM users visited clinics and less than 10% CM hospitals.

Based on the ICD-9-CM code, we found that diseases of the respiratory system, diseases of the musculoskeletal system and connective tissue, injury and poisoning, signs, symptoms and ill-defined conditions, and diseases of the digestive system were the primary indications in CM. Similar patterns have been reported previously [12,14]. However, the top two primary indications were also the same as those in WM. We have also found that the utilization of these two health-care services was higher in winter than in the remaining seasons. These findings may be due to the fact that diseases of the respiratory system occurs more frequency in winter. These findings indicate that CM may be a feasible complementary choice for the patients with respiratory diseases.

Herbal medication is the major component of CM accounting for more than two-thirds of the ambulatory visits. In the United States, herbal products have an annual market of US$5.1 billion [41] and 38.2 million adults have used herbs and supplements [42]. The herbal prescriptions in the Western countries are usually with a single herb whereas the majority of prescriptions in Taiwan are composed of 3–6 herbs and are often prescribed for three times a day [17]. Under the NHI program, herbal formulas were provided as standard pharmaceutical products in standard powder forms. This strategy not only can control the quality of the prescriptions but also enable the standardization of herbal formulas. Acupuncture is another popular form of CAM [3-7]. It is mainly employed in the treatment of neurologic and musculoskeletal diseases [18,43,44]. In Taiwan, about 23% of people used acupuncture from 1996 to 2002 [18]. We obtained similar results in this study. In addition to an increasing trend in the utilization of acupuncture, the prevalence of muscle strain therapy was also increased in recent years. This increase may be due to the increase in the number of providers [13]. However, further investigations are needed to confirm this suggestion.

This study is the first population-based investigation to determine the utilization patterns of CM and WM in Taiwan under the NHI Program. Since we obtained the data from a sufficiently large national representative sample from the NHI sample files, some of the common shortcomings in interview or questionnaire surveys may be avoided. The large sample size and the comprehensive datasets allow us to study a wide array of factors in the general population. However, we have excluded enrolees who were newborns aged below 2 years in 1997, those died in the study period, aliens, those with incomplete data, and those without continuous enrolment information. This manipulation of the data may lead to some selection bias. Since we used only the NHI claims data, we are unable to determine the utilization of CM services not covered by the NHI program. Therefore, the utilization of CM in Taiwan might be underestimated.

## Conclusion

The utilization of CAM has rapidly increased in many countries during the last two decades [4-7]. CAM is rarely covered in national health systems. However, 75% of the Dutch population wanted hospitals to provide CAM [45] and 74% of the people in UK felt that complementary therapies should be available on the national health system [46]. In Taiwan, the NHI Program is a comprehensive and universal health insurance program. This program not only covers conventional WM services, but also traditional CM services. Moreover, CM is popular and more than 60% of all beneficiaries of this health insurance system had used CM at least once a year [19]. In this study, we found that the rate of increase in the use of CM was smaller than that in WM (Table 2) and that the increase was not apparently shown in the number of visits per user. Taiwan's experience of covering CM services under its national health insurance system may serve as an important reference to other countries. This strategy offers people another choice for medical care services with mandated coverage.

## Competing interests

The authors declare that they have no competing interests.

## Authors' contributions

LCC, NH, YJC, CHL and YTH contributed equally to this paper. FYK carried out the analysis. LCC, NH, YJC, CHL, FYK and HYT pictured the idea and drafted the manuscript. All authors have read and approved the final manuscript.

## Pre-publication history

The pre-publication history for this paper can be accessed here:


